# Lupus Erythematosus Tumidus in the Differential Diagnosis of Facial Erythema

**DOI:** 10.7759/cureus.108152

**Published:** 2026-05-02

**Authors:** Zaira P Sánchez Albarrán, Francisco J Lugo-Rincon Gallardo, María del Mar Vargas Rosales, Zoila A Castañón Martínez, Miguel A Dorantes Solis

**Affiliations:** 1 Internal Medicine, Hospital Regional de Alta Especialidad del Bajío, León, MEX; 2 Dermatology, Hospital General de México “Dr. Eduardo Liceaga”, Mexico City, MEX

**Keywords:** cutaneous lupus, facial erythema, lupus tumidus, photosensitivity, skin biopsy

## Abstract

Lupus erythematosus is an autoimmune disease that frequently presents with dermatologic manifestations. When it is limited to the facial area, it is difficult to distinguish from other inflammatory diseases. Among the differential diagnoses of facial erythema, lupus erythematosus tumidus (LET) is an uncommon entity that represents a subtype of lupus erythematosus. It is characterized as a photosensitive dermatosis with a favorable clinical course and prognosis. A 32-year-old female patient presented with dermatosis localized to the face, specifically in the frontal and maxillary regions, characterized by persistent facial erythema with papular eruptions over a two-year period. Initially diagnosed as rosacea, she received multiple treatments without clinical improvement. Subsequently, histopathological findings led to the diagnosis of LET, a form of intermittent cutaneous lupus erythematosus that occurs much less frequently than other forms of cutaneous lupus. It presents with a relapsing course associated with sun exposure, histopathologically characterized by superficial and deep perivascular and periadnexal lymphocytic infiltrates with dermal mucin deposition. It may clinically mimic other skin disorders. Therefore, skin biopsies are indicated in various clinical scenarios when histopathologic information is required to make a diagnosis and establish the definitive treatment plan.

## Introduction

Facial inflammatory diseases manifest primarily as facial erythema, papules, and pustules. Among the most common causes are rosacea, seborrheic dermatitis, contact dermatitis, atopic dermatitis, and acne. However, the diagnosis of these diseases is mostly symptomatic, which can lead to confusion and result in inaccurate diagnoses and treatments [1]. In patients with facial erythema, it is necessary to consider autoimmune diseases, such as lupus erythematosus. A skin biopsy could reveal specific features that guide the diagnosis [1].

Lupus erythematosus is an autoimmune disease that encompasses a wide spectrum of clinical and histological manifestations. Cutaneous lesions constitute one of the most frequent clinical findings, as up to 85% of cases exhibit dermatological activity [2]. When these are limited to the facial area, especially in early stages, it is difficult to distinguish them from other inflammatory diseases. Lupus erythematosus tumidus (LET) is a variant of cutaneous lupus erythematosus characterized by erythematous, infiltrated plaques with a smooth surface, marked photosensitivity, and resolution without scarring. It is frequently misdiagnosed as rosacea due to the predominance of facial erythema; however, unlike rosacea, LET presents with firm lesions and typically lacks pustules. Facial erythema necessitates an extensive differential diagnosis, where its characteristics and systemic manifestations guide the etiology. This may present as a pivotal symptom of autoimmune diseases, such as lupus erythematosus, whose cutaneous manifestations can mimic rosacea. In patients with facial erythema who do not respond to rosacea treatment regimens, it is necessary to perform serological tests and a skin biopsy for differential diagnosis [3].

## Case presentation

**Figure 1:**
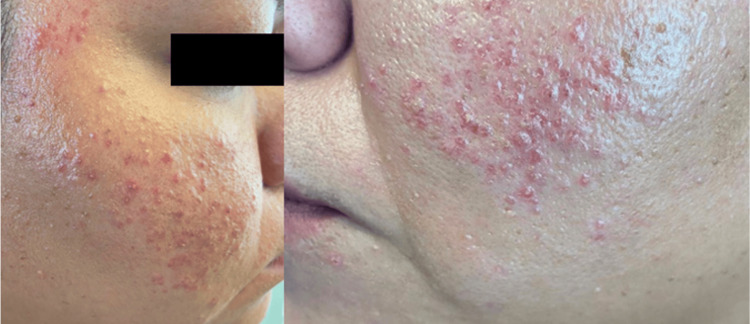
Lateral views Papular eruption in the maxillary region.

**Figure 2:**
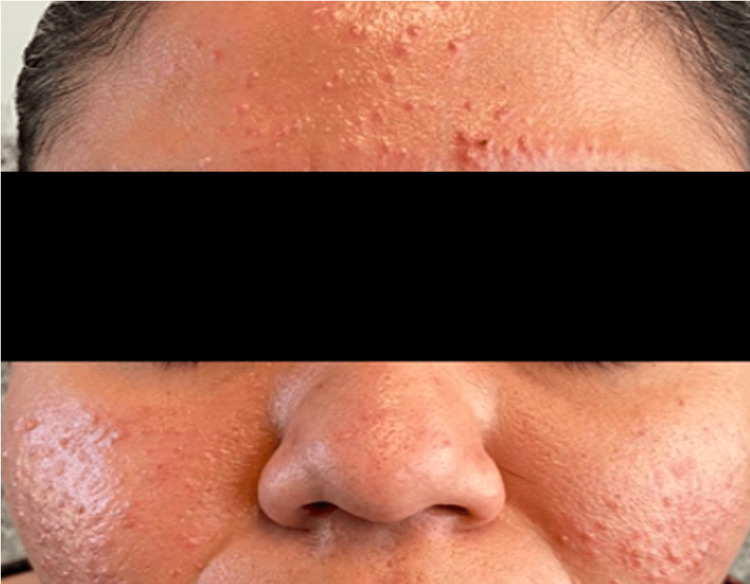
Anterior view Erythema with a papular eruption predominantly in the frontal and maxillary regions, sparing the nasolabial folds. Erythema masked by makeup.

A 32-year-old female patient with a past medical history significant for asthma and hypothyroidism presented with a two-year history of recurrent dermatosis localized to the face, predominantly involving the frontal and maxillary regions. The lesions were described as multiple erythematous, non-scarring, pruritic papules and plaques that appeared in intermittent flares. The patient reported a clear association between sun exposure and lesion exacerbation. Cutaneous lesions typically appeared within 24-48 hours following ultraviolet exposure and could persist for several days, consistent with the well-known photosensitive nature of LET (Figure 1, Figure 2). The patient denied systemic symptoms such as fever, arthralgias, or fatigue. A comprehensive serologic evaluation was performed to assess for underlying autoimmune disease. Antinuclear antibodies were negative.

Initially, the condition was clinically diagnosed as rosacea, and the patient received conventional treatment; however, she experienced persistence and occasional exacerbation of the lesions, with no significant clinical improvement. Due to the atypical course and poor therapeutic response, a skin biopsy was performed.

**Figure 3:**
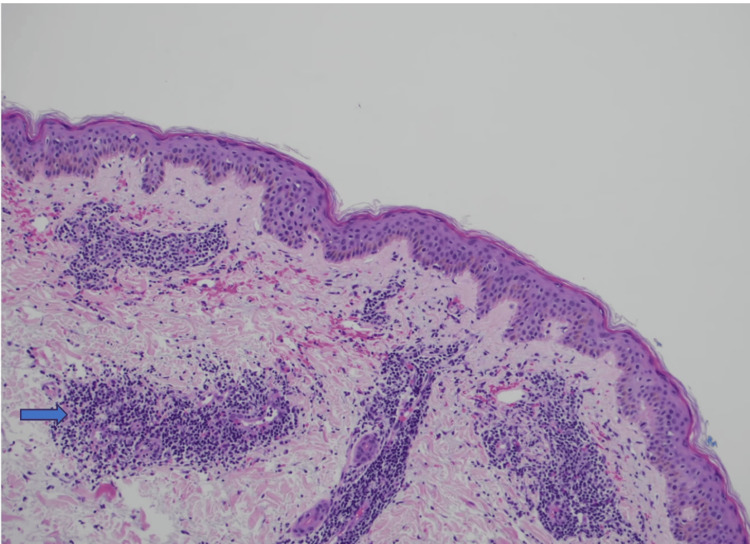
Skin biopsy Perivascular and periadnexal lymphocytic infiltration in the superficial and deep dermis.

**Figure 4:**
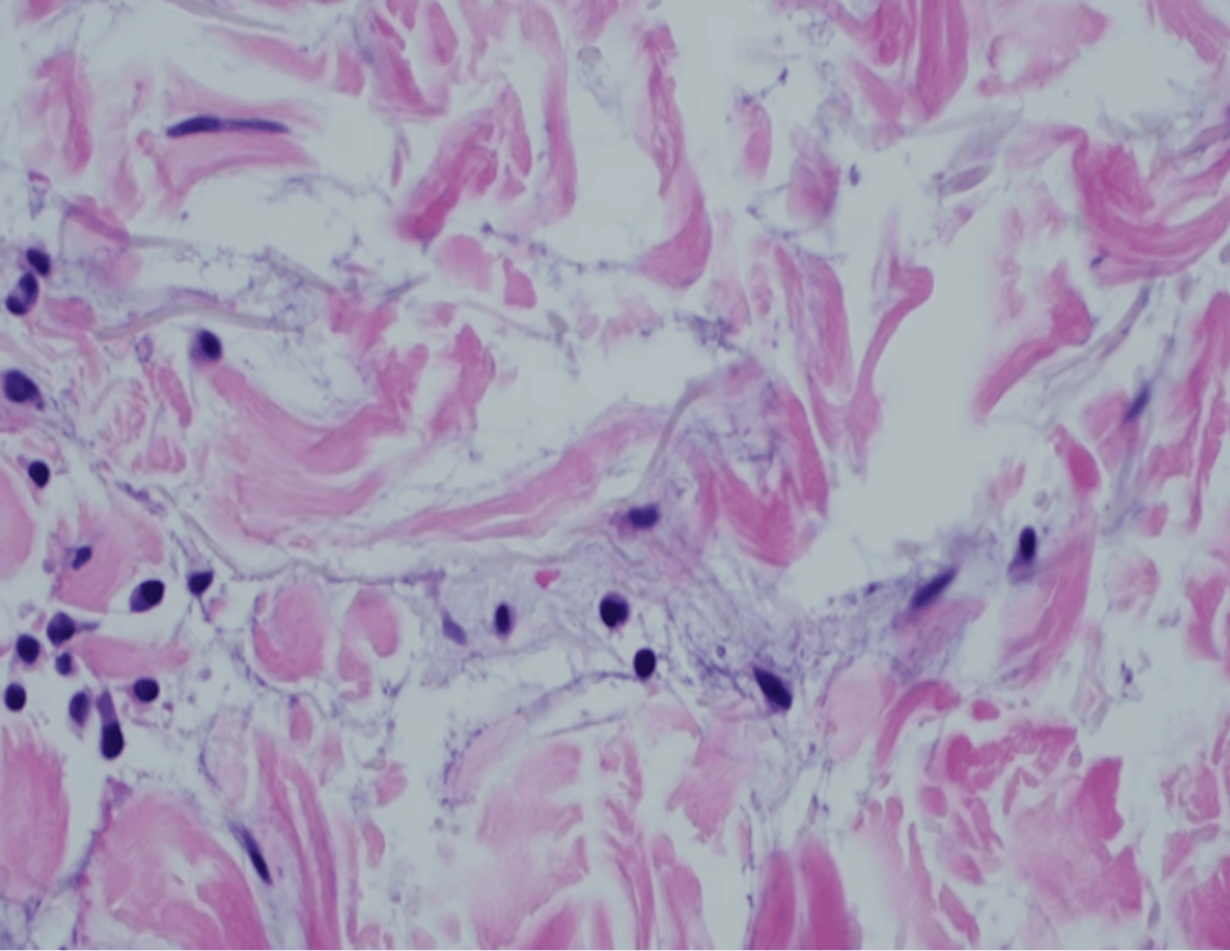
Skin biopsy Diffuse mucin deposition is observed.

Histopathological examination revealed a dense lymphocytic inflammatory infiltrate distributed in a perivascular and periadnexal pattern involving both the superficial and deep dermis (Figure 3). Additionally, abundant mucin deposition within the dermis was observed (Figure 4). The epidermis remained largely preserved, without significant interface changes. These findings were consistent with a diagnosis of LET.

Several conditions were considered and actively ruled out based on clinical and histopathological findings. Rosacea was considered due to the centrofacial erythema; however, the absence of pustules, telangiectasia, and lack of response to standard therapies argued against this diagnosis. Seborrheic dermatitis was excluded due to the absence of greasy scaling and its typical distribution. Polymorphic light eruption was considered, given the photosensitivity, but the chronicity and morphology of the lesions were not consistent. Jessner’s lymphocytic infiltrate was also included in the differential; however, the presence of dermal mucin deposition on histopathology favored LET (Table 1).

**Table 1:** Differential diagnosis of facial erythema ANA, antinuclear antibody; LET, lupus erythematosus tumidus

Feature	LET	Rosacea	Polymorphic light eruption
Lesion morphology	Smooth, erythematous, infiltrated plaques; no scaling	Erythema with papules and pustules	Papules, vesicles, or plaques
Consistency	Firm, indurated	Soft	Variable
Photosensitivity	Marked lesions appear 24-48 hours after UV exposure	May flare with triggers (sun, heat), but not delayed	Strong; typically hours to days after sun exposure
Distribution	Face, neck, upper trunk	Centrofacial (cheeks, nose, chin, forehead)	Sun-exposed areas (arms, chest, neck)
Pustules	Absent	Common	Absent
Course	Chronic, recurrent; heals without scarring	Chronic with flares	Seasonal, recurrent
Histopathology	Perivascular/periadnexal lymphocytic infiltrate + dermal mucin; epidermis spared	Vascular dilation, perifollicular inflammation	Superficial perivascular lymphocytic infiltrate; no mucin
Serology	Usually negative (ANA often negative)	Negative	Negative
Treatment response	Antimalarials (e.g., hydroxychloroquine)	Topical/oral antibiotics, ivermectin	Sun avoidance, topical steroids

Based on the clinical and histological correlation, the patient was started on hydroxychloroquine therapy. Adjunctive measures, including strict photoprotection, were also recommended. Following initiation of treatment, the patient showed progressive improvement, with a reduction in erythema, pruritus, and number of lesions during follow-up.

## Discussion

LET is a rare photosensitive dermatosis that was previously considered a subtype of chronic cutaneous lupus erythematosus. However, due to its particular clinical evolution and favorable prognosis, it has been reclassified into a distinct category known as intermittent cutaneous lupus erythematosus [4].

The term “lupus erythematosus tumidus” was introduced in 1909 by the German dermatologist Erich Hoffmann [5,6]. It affects men and women in similar proportions, with an average age of diagnosis of 34.6 and 38.5 years [6]. Approximately 20% of patients with LET present positive antinuclear antibody titers. In addition to cutaneous manifestations, nearly 50% of patients experience symptoms such as pruritus, pain sensitivity, and photosensitivity [6].

Lesions found in LET consist of erythematous papules, plaques, or annular lesions with a succulent appearance that predominate in sun-exposed areas such as the neckline, shoulders, face, and arms. These lesions usually occur in outbreaks, almost always related to sun exposure, with a latency period that can vary from 24 hours to a week. They do not present scaling, follicular plugging, or atrophy, features that help differentiate it from other forms of cutaneous lupus [6].

From a histological point of view, LET is characterized by a lymphocytic infiltrate surrounding vascular and adnexal structures in both the superficial and deep dermis, accompanied by abundant mucin deposits. The epidermis, in general, remains without alterations [7,8]. In some cases, there may be minimal alterations of the epidermis or the dermoepidermal junction, such as mild vacuolar degeneration, or even an absence of mucin deposits; however, this does not exclude the diagnosis [7]. Direct immunofluorescence (DIF) studies performed on skin samples affected by LET usually show negative results for immunoglobulin deposits and complement components [9]. Differential diagnoses include pathologies such as erythema nodosum, Jessner’s lymphocytic infiltrate, and reticular erythematous mucinosis [10,11].

Initial treatment includes topical corticosteroids and photoprotection, with resolution of lesions in a variable proportion of patients (up to 45-80% in some studies). In refractory cases, antimalarials (chloroquine or hydroxychloroquine) are highly effective, achieving significant improvement in most patients within a few weeks [12]. Cases that do not respond to first-line treatment may benefit from alternative therapies such as methotrexate, thalidomide, or deucravacitinib [13].

From a diagnostic standpoint, this case was initially distinguished from rosacea by the lack of response to conventional therapy, as well as the absence of key clinical features such as pustules and telangiectasia, and the presence of firm, infiltrated plaques. Additionally, the histopathological findings were not compatible with rosacea and instead supported the diagnosis of LET. In this context, dermal mucin deposition represents a key distinguishing feature, particularly when differentiating LET from entities such as Jessner’s lymphocytic infiltrate, in which mucin is typically absent or minimal. Finally, DIF was not performed in this case, which constitutes a limitation of the study; however, clinicopathological correlation was sufficient to establish the diagnosis.

## Conclusions

Facial erythema represents a diagnostic challenge due to its multiple etiologies; therefore, in patients refractory to treatment, a biopsy is justified. Failure to consider LET early in the diagnostic approach may lead to prolonged misdiagnosis and unnecessary treatments. In the case presented above, the biopsy was crucial, as it allowed a definitive diagnosis to be established and targeted therapy to be initiated. Furthermore, it is important to highlight that LET is an uncommon entity in the differential diagnosis of facial erythema, which contributes to its frequent under-recognition. It is characterized by an intermittent clinical course and an association with sun exposure, both of which were evidenced in this patient. Once treatment was initiated, there was a notable improvement in symptoms, confirming the favorable prognosis of this entity.
